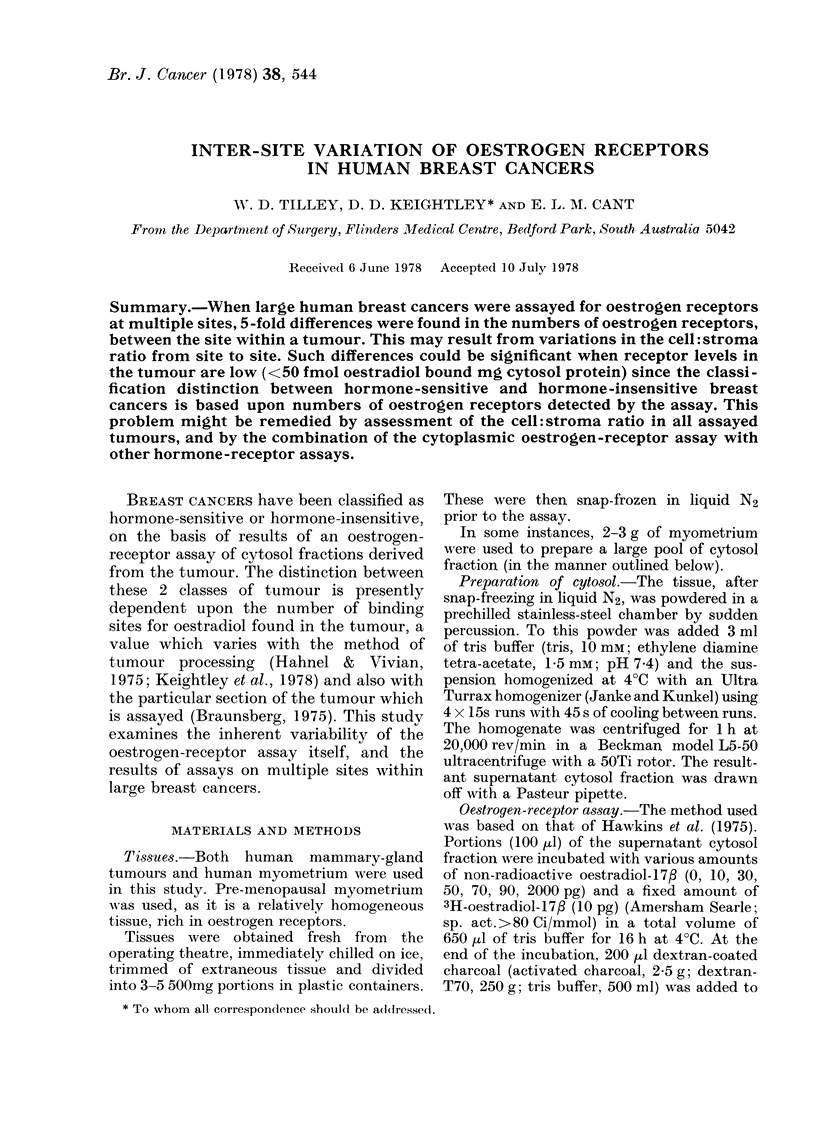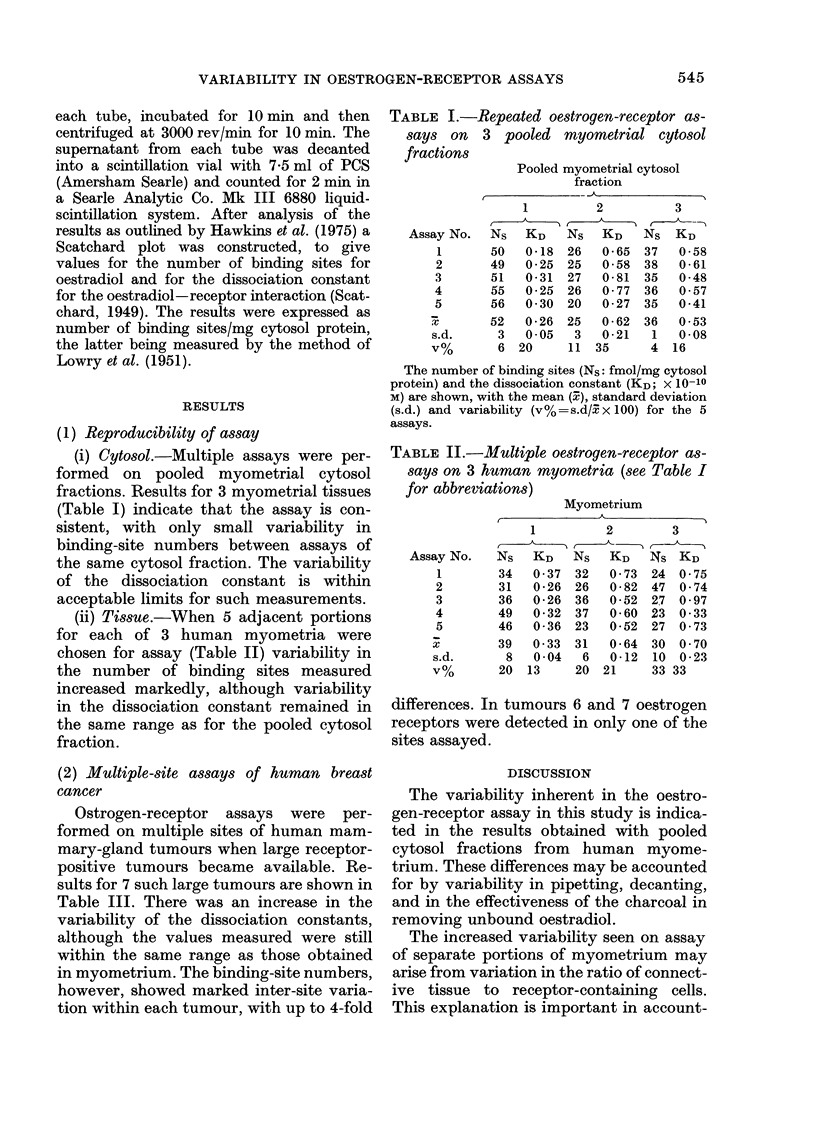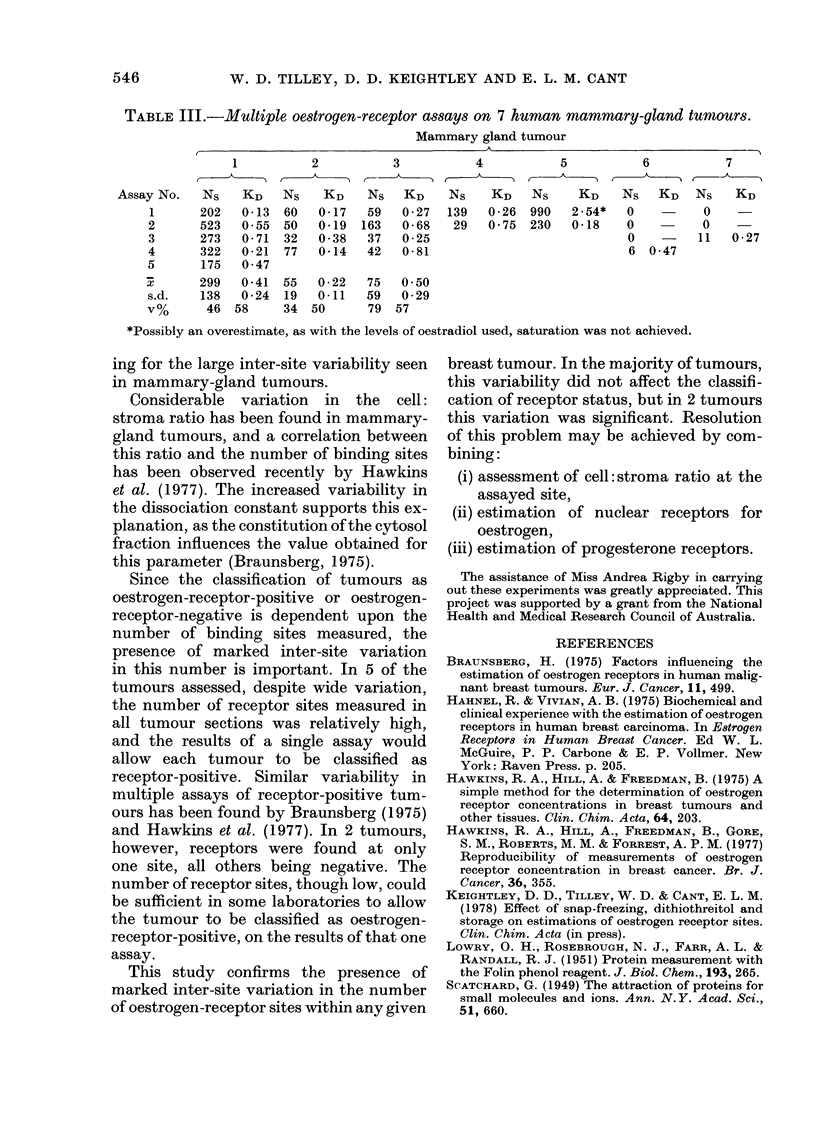# Inter-site variation of oestrogen receptors in human breast cancers.

**DOI:** 10.1038/bjc.1978.242

**Published:** 1978-10

**Authors:** W. D. Tilley, D. D. Keightley, E. L. Cant

## Abstract

When large human breast cancers were assayed for oestrogen receptors at multiple sites, 5-fold differences were found in the numbers of oestrogen receptors, between the site within a tumour. This may result from variations in the cell:stroma ratio from site to site. Such differences could be significant when receptor levels in the tumour are low (less than 50 fmol oestradiol bound mg cytosol protein) since the classification distinction between hormone-sensitive and hormone-insensitive breast cancers is based upon numbers of oestrogen receptors detected by the assay. This problem might be remedied by assessment of the cell:stroma ratio in all assayed tumours, and by the combination of the cytoplasmic oestrogen-receptor assay with other hormone-receptor assays.


*Br. J. Cancer* (1978) **38**, 544

### INTER-SITE VARIATION OF OESTROGEN RECEPTORS IN HUMAN BREAST CANCERS

W. D. TILLEY, D. D. KEIGHTLEY* AND E. L. M. CANT

*From the Department of Surgery, Flinders Medical Centre, Bedford Park, South Australia* 5042

Received 6 June 1978 Accepted 10 July 1978

**Summary.—When large human breast cancers were assayed for oestrogen receptors at multiple sites, 5-fold differences were found in the numbers of oestrogen receptors, between the site within a tumour. This may result from variations in the cell:stroma ratio from site to site. Such differences could be significant when receptor levels in the tumour are low (<50 fmol oestradiol bound mg cytosol protein) since the classification distinction between hormone-sensitive and hormone-insensitive breast cancers is based upon numbers of oestrogen receptors detected by the assay. This problem might be remedied by assessment of the cell:stroma ratio in all assayed tumours, and by the combination of the cytoplasmic oestrogen-receptor assay with other hormone-receptor assays.**

BREAST CANCERS have been classified as hormone-sensitive or hormone-insensitive, on the basis of results of an oestrogen-receptor assay of cytosol fractions derived from the tumour. The distinction between these 2 classes of tumour is presently dependent upon the number of binding sites for oestradiol found in the tumour, a value which varies with the method of tumour processing (Hahnel & Vivian, 1975; Keightley *et al.*, 1978) and also with the particular section of the tumour which is assayed (Braunsberg, 1975). This study examines the inherent variability of the oestrogen-receptor assay itself, and the results of assays on multiple sites within large breast cancers.

### MATERIALS AND METHODS

*Tissues.*—Both human mammary-gland tumours and human myometrium were used in this study. Pre-menopausal myometrium was used, as it is a relatively homogeneous tissue, rich in oestrogen receptors.

Tissues were obtained fresh from the operating theatre, immediately chilled on ice, trimmed of extraneous tissue and divided into 3–5 500mg portions in plastic containers. These were then snap-frozen in liquid $N_2$ prior to the assay.

In some instances, 2–3 g of myometrium were used to prepare a large pool of cytosol fraction (in the manner outlined below).

*Preparation of cytosol.*—The tissue, after snap-freezing in liquid $N_2$, was powdered in a prechilled stainless-steel chamber by sudden percussion. To this powder was added 3 ml of tris buffer (tris, 10 mM; ethylene diamine tetra-acetate, 1·5 mM; pH 7·4) and the suspension homogenized at 4°C with an Ultra Turrax homogenizer (Janke and Kunkel) using 4 × 15s runs with 45 s of cooling between runs. The homogenate was centrifuged for 1 h at 20,000 rev/min in a Beckman model L5-50 ultracentrifuge with a 50Ti rotor. The resultant supernatant cytosol fraction was drawn off with a Pasteur pipette.

*Oestrogen-receptor assay.*—The method used was based on that of Hawkins *et al.* (1975). Portions (100 μl) of the supernatant cytosol fraction were incubated with various amounts of non-radioactive oestradiol-17β (0, 10, 30, 50, 70, 90, 2000 pg) and a fixed amount of $^3$H-oestradiol-17β (10 pg) (Amersham Searle; sp. act.>80 Ci/mmol) in a total volume of 650 μl of tris buffer for 16 h at 4°C. At the end of the incubation, 200 μl dextran-coated charcoal (activated charcoal, 2·5 g; dextran-T70, 250 g; tris buffer, 500 ml) was added to

* To whom all correspondence should be addressed.

each tube, incubated for 10 min and then centrifuged at 3000 rev/min for 10 min. The supernatant from each tube was decanted into a scintillation vial with 7·5 ml of PCS (Amersham Searle) and counted for 2 min in a Searle Analytic Co. Mk III 6880 liquid-scintillation system. After analysis of the results as outlined by Hawkins *et al.* (1975) a Scatchard plot was constructed, to give values for the number of binding sites for oestradiol and for the dissociation constant for the oestradiol—receptor interaction (Scatchard, 1949). The results were expressed as number of binding sites/mg cytosol protein, the latter being measured by the method of Lowry *et al.* (1951).

### RESULTS

### (1) *Reproducibility of assay*

(i) *Cytosol.*—Multiple assays were performed on pooled myometrial cytosol fractions. Results for 3 myometrial tissues (Table I) indicate that the assay is consistent, with only small variability in binding-site numbers between assays of the same cytosol fraction. The variability of the dissociation constant is within acceptable limits for such measurements.

(ii) *Tissue.*—When 5 adjacent portions for each of 3 human myometria were chosen for assay (Table II) variability in the number of binding sites measured increased markedly, although variability in the dissociation constant remained in the same range as for the pooled cytosol fraction.

### (2) *Multiple-site assays of human breast cancer*

Ostrogen-receptor assays were performed on multiple sites of human mammary-gland tumours when large receptor-positive tumours became available. Results for 7 such large tumours are shown in Table III. There was an increase in the variability of the dissociation constants, although the values measured were still within the same range as those obtained in myometrium. The binding-site numbers, however, showed marked inter-site variation within each tumour, with up to 4-fold differences. In tumours 6 and 7 oestrogen receptors were detected in only one of the sites assayed.

TABLE I.—*Repeated oestrogen-receptor assays on 3 pooled myometrial cytosol fractions*

| | Pooled myometrial cytosol fraction | | | | | |
|---|---|---|---|---|---|---|
| | 1 | | 2 | | 3 | |
| Assay No. | $N_S$ | $K_D$ | $N_S$ | $K_D$ | $N_S$ | $K_D$ |
| 1 | 50 | 0·18 | 26 | 0·65 | 37 | 0·58 |
| 2 | 49 | 0·25 | 25 | 0·58 | 38 | 0·61 |
| 3 | 51 | 0·31 | 27 | 0·81 | 35 | 0·48 |
| 4 | 55 | 0·25 | 26 | 0·77 | 36 | 0·57 |
| 5 | 56 | 0·30 | 20 | 0·27 | 35 | 0·41 |
| $\bar{x}$ | 52 | 0·26 | 25 | 0·62 | 36 | 0·53 |
| s.d. | 3 | 0·05 | 3 | 0·21 | 1 | 0·08 |
| v% | 6 | 20 | 11 | 35 | 4 | 16 |

The number of binding sites ($N_S$: fmol/mg cytosol protein) and the dissociation constant ($K_D$; $\times 10^{-10}$ M) are shown, with the mean ($\bar{x}$), standard deviation (s.d.) and variability (v% = s.d/$\bar{x} \times 100$) for the 5 assays.

TABLE II.—*Multiple oestrogen-receptor assays on 3 human myometria (see Table I for abbreviations)*

| | Myometrium | | | | | |
|---|---|---|---|---|---|---|
| | 1 | | 2 | | 3 | |
| Assay No. | $N_S$ | $K_D$ | $N_S$ | $K_D$ | $N_S$ | $K_D$ |
| 1 | 34 | 0·37 | 32 | 0·73 | 24 | 0·75 |
| 2 | 31 | 0·26 | 26 | 0·82 | 47 | 0·74 |
| 3 | 36 | 0·26 | 36 | 0·52 | 27 | 0·97 |
| 4 | 49 | 0·32 | 37 | 0·60 | 23 | 0·33 |
| 5 | 46 | 0·36 | 23 | 0·52 | 27 | 0·73 |
| $\bar{x}$ | 39 | 0·33 | 31 | 0·64 | 30 | 0·70 |
| s.d. | 8 | 0·04 | 6 | 0·12 | 10 | 0·23 |
| v% | 20 | 13 | 20 | 21 | 33 | 33 |

### DISCUSSION

The variability inherent in the oestrogen-receptor assay in this study is indicated in the results obtained with pooled cytosol fractions from human myometrium. These differences may be accounted for by variability in pipetting, decanting, and in the effectiveness of the charcoal in removing unbound oestradiol.

The increased variability seen on assay of separate portions of myometrium may arise from variation in the ratio of connective tissue to receptor-containing cells. This explanation is important in account-

TABLE III.—*Multiple oestrogen-receptor assays on 7 human mammary-gland tumours.*

| | Mammary gland tumour | | | | | | | | | | | | | |
|---|---|---|---|---|---|---|---|---|---|---|---|---|---|---|
| | 1 | | 2 | | 3 | | 4 | | 5 | | 6 | | 7 | |
| Assay No. | $N_S$ | $K_D$ | $N_S$ | $K_D$ | $N_S$ | $K_D$ | $N_S$ | $K_D$ | $N_S$ | $K_D$ | $N_S$ | $K_D$ | $N_S$ | $K_D$ |
| 1 | 202 | 0·13 | 60 | 0·17 | 59 | 0·27 | 139 | 0·26 | 990 | 2·54* | 0 | — | 0 | — |
| 2 | 523 | 0·55 | 50 | 0·19 | 163 | 0·68 | 29 | 0·75 | 230 | 0·18 | 0 | — | 0 | — |
| 3 | 273 | 0·71 | 32 | 0·38 | 37 | 0·25 | | | | | 0 | — | 11 | 0·27 |
| 4 | 322 | 0·21 | 77 | 0·14 | 42 | 0·81 | | | | | 6 | 0·47 | | |
| 5 | 175 | 0·47 | | | | | | | | | | | | |
| $\bar{x}$ | 299 | 0·41 | 55 | 0·22 | 75 | 0·50 | | | | | | | | |
| s.d. | 138 | 0·24 | 19 | 0·11 | 59 | 0·29 | | | | | | | | |
| v% | 46 | 58 | 34 | 50 | 79 | 57 | | | | | | | | |

*Possibly an overestimate, as with the levels of oestradiol used, saturation was not achieved.

ing for the large inter-site variability seen in mammary-gland tumours.

Considerable variation in the cell: stroma ratio has been found in mammary-gland tumours, and a correlation between this ratio and the number of binding sites has been observed recently by Hawkins *et al.* (1977). The increased variability in the dissociation constant supports this explanation, as the constitution of the cytosol fraction influences the value obtained for this parameter (Braunsberg, 1975).

Since the classification of tumours as oestrogen-receptor-positive or oestrogen-receptor-negative is dependent upon the number of binding sites measured, the presence of marked inter-site variation in this number is important. In 5 of the tumours assessed, despite wide variation, the number of receptor sites measured in all tumour sections was relatively high, and the results of a single assay would allow each tumour to be classified as receptor-positive. Similar variability in multiple assays of receptor-positive tumours has been found by Braunsberg (1975) and Hawkins *et al.* (1977). In 2 tumours, however, receptors were found at only one site, all others being negative. The number of receptor sites, though low, could be sufficient in some laboratories to allow the tumour to be classified as oestrogen-receptor-positive, on the results of that one assay.

This study confirms the presence of marked inter-site variation in the number of oestrogen-receptor sites within any given breast tumour. In the majority of tumours, this variability did not affect the classification of receptor status, but in 2 tumours this variation was significant. Resolution of this problem may be achieved by combining:

(i) assessment of cell:stroma ratio at the assayed site,
(ii) estimation of nuclear receptors for oestrogen,
(iii) estimation of progesterone receptors.

The assistance of Miss Andrea Rigby in carrying out these experiments was greatly appreciated. This project was supported by a grant from the National Health and Medical Research Council of Australia.

### REFERENCES

BRAUNSBERG, H. (1975) Factors influencing the estimation of oestrogen receptors in human malignant breast tumours. *Eur. J. Cancer*, **11**, 499.

HAHNEL, R. & VIVIAN, A. B. (1975) Biochemical and clinical experience with the estimation of oestrogen receptors in human breast carcinoma. In *Estrogen Receptors in Human Breast Cancer*. Ed W. L. McGuire, P. P. Carbone & E. P. Vollmer. New York: Raven Press. p. 205.

HAWKINS, R. A., HILL, A. & FREEDMAN, B. (1975) A simple method for the determination of oestrogen receptor concentrations in breast tumours and other tissues. *Clin. Chim. Acta*, **64**, 203.

HAWKINS, R. A., HILL, A., FREEDMAN, B., GORE, S. M., ROBERTS, M. M. & FORREST, A. P. M. (1977) Reproducibility of measurements of oestrogen receptor concentration in breast cancer. *Br. J. Cancer*, **36**, 355.

KEIGHTLEY, D. D., TILLEY, W. D. & CANT, E. L. M. (1978) Effect of snap-freezing, dithiothreitol and storage on estimations of oestrogen receptor sites. *Clin. Chim. Acta* (in press).

LOWRY, O. H., ROSEBROUGH, N. J., FARR, A. L. & RANDALL, R. J. (1951) Protein measurement with the Folin phenol reagent. *J. Biol. Chem.*, **193**, 265.

SCATCHARD, G. (1949) The attraction of proteins for small molecules and ions. *Ann. N.Y. Acad. Sci.*, **51**, 660.